# A Case of Crigler-Najjar Syndrome Type II During Pregnancy and Its Management

**DOI:** 10.7759/cureus.59075

**Published:** 2024-04-26

**Authors:** Sukanya Singh, Surekha Tayade, Nidhi Makhija, Drashti Patel, Akanksha Singh

**Affiliations:** 1 Department of Obstetrics and Gynaecology, Jawaharlal Nehru Medical College, Datta Meghe Institute of Medical Sciences, Wardha, IND; 2 Department of Obstetrics and Gynaecology, Institute of Post Graduate Medical Education, Research and Seth Sukhlal Karnani Memorial Hospital, Kolkata, IND

**Keywords:** hyperbilirubinemia, ugt1a1 gene, phenobarbitone, uridine 5-diphosphate glucuronosyl transferase enzyme, maternal crigler-najjar syndrome

## Abstract

Crigler-Najjar syndrome (CNS) is a genetic syndrome that results in increased levels of unconjugated bilirubin due to less or completely nonfunctional enzyme, uridine diphosphoglucoronyltransferase (UDPGT) in hepatocytes. When bilirubin metabolism is compromised, hyperbilirubinemia is caused, which results in increased levels of unconjugated and conjugated bilirubin in the bloodstream. CNS is an autosomal recessive disorder, usually noticeable as people get older. This disorder is divided into two types: CNS type I and CNS type II, which are caused by homozygous or compound heterozygous mutations in the UDP glucuronosyltransferase family 1 member A1 (UGT1A1) gene. The disorder affects all races and genders equally, with a prevalence of one per million births. CNS type I is more severe and has almost undetectable UDPGT expression activity, and affected individuals die before one year of age. Consanguineous marriages are a major risk factor as CNS is inherited in an autosomal recessive manner. Being rare, maternal CNS type II is yet to be completely understood in terms of its impact on the mother, her pregnancy, and the infant. We aim to present a case of a pregnant female with CNS type II and its clinical course. She was monitored closely during her pregnancy. The treatment protocol was followed as per previously reported cases and was managed on low, non-teratogenic doses of phenobarbitone. A successful outcome with the birth of a healthy infant having normal neurological development till six months follow-up was observed.

## Introduction

Crigler-Najjar syndrome (CNS) during pregnancy has elevated levels of unconjugated bilirubin both in the mother and neonate. Increased bilirubin levels in the long term can impair the neurological development of the newborn due to poor uridine di-phosphoglucuronate glucuronosyltransferase (UDPGT) activity making timely diagnosis crucial and initiation of proper therapeutic management. A greater level of unconjugated bilirubin in newborns can lead to kernicterus, which can sometimes even result in sudden death [1]. CNS has been differentiated into two types: type I and type II based on complete and incomplete deficiency of hepatic UDPGT, respectively, which is responsible for bilirubin excretion and conjugation [2,3]. Normal hepatic UDPGT levels are <10% and serum ranges are between 3 and 20 mg/dL [4]. In this case, we aim to present a disease course with a successful outcome in a CNS type II pregnancy.

## Case presentation

**Figure 1:**
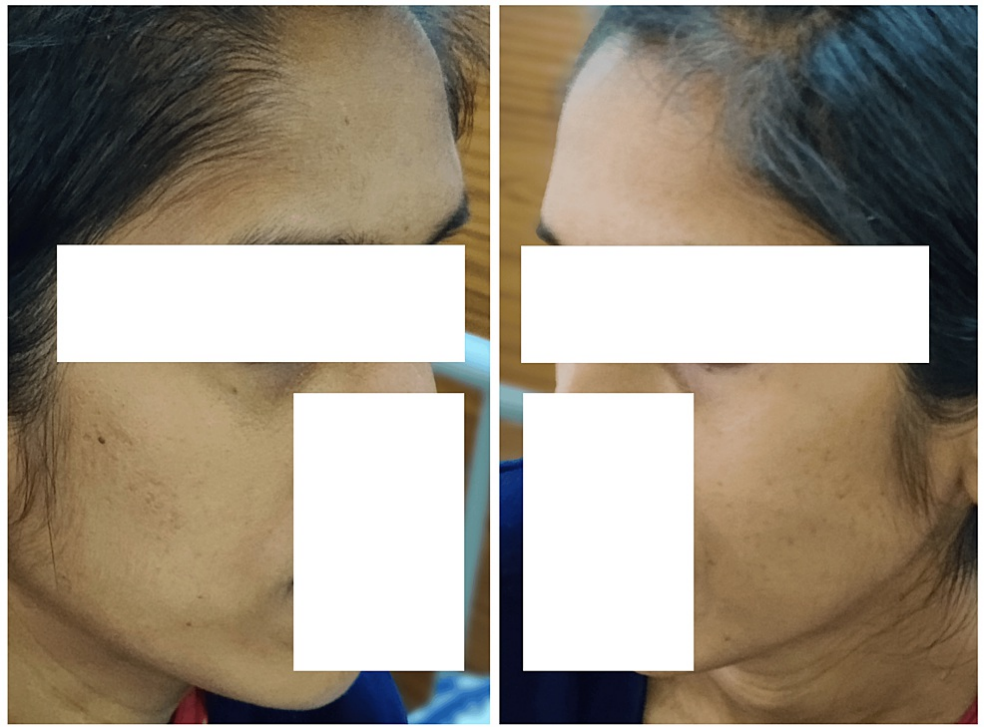
Left view and right view of the face showing pale and icteric skin

**Figure 2:**
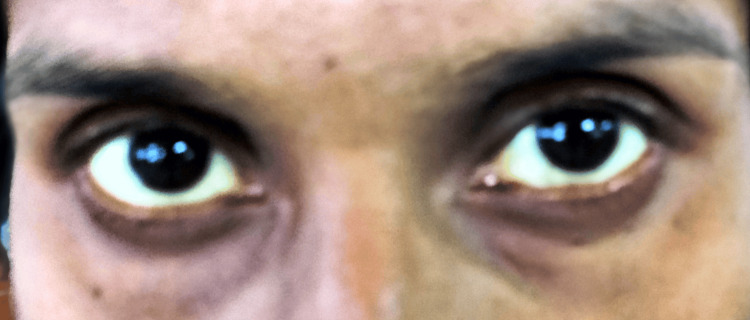
Icterus visible on bulbar conjunctiva

A 33-year-old pregnant woman of Indian origin with 37 weeks and six days gestation with a positive history of jaundice without hemolytic anemia presented at our hospital. This was her second pregnancy with one live child. She visited the outpatient department (OPD) of Acharya Vinoba Bhave Hospital, during the first trimester of her pregnancy due to unmanaged jaundice (since two weeks) with pale and icteric skin and icterus visible on bulbar conjunctiva (Figure 1, 2).

**Figure 3:**
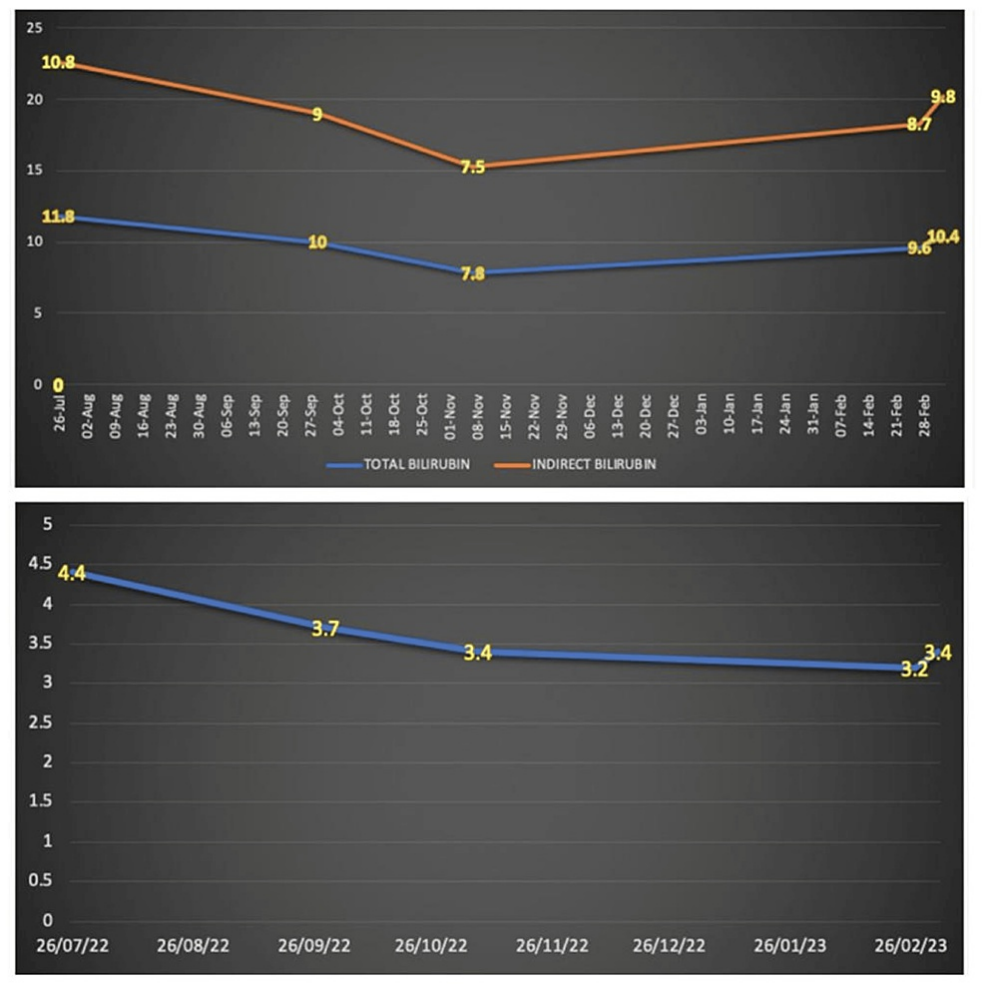
Patients’ total bilirubin, indirect bilirubin, and albumin levels during the course of her pregnancy Top panel: total bilirubin and indirect bilirubin levels (in mg/dL) levels; bottom panel: indicative of albumin levels (in mg/dL)

The patient had dark yellow urine with yellow discoloration of sclera since birth and no neurological abnormalities. She had a similar history in her last pregnancy as well, with no available medical records. The patient's blood group was B Rh "positive" and the husband's blood group was A Rh "positive" with no associated family history. On OPD visits, clinical examination and transabdominal ultrasound were carried out at the eighth, 12th, 21st, and 25th weeks, which revealed adequate liquor and a single intrauterine live fetus with placenta developing anteriorly. Fetal weight increased progressively throughout the trimesters. The patient's blood reports showed raised bilirubin levels on the Vitros 5600 integrated system; total and direct bilirubin has been measured by commercially available Diazo-based colorimetric assays. Total albumin, bilirubin, and indirect bilirubin at eight weeks gestation were 4.4, 11.8, and 10.8 mg/dL, respectively, with normal liver enzymes. Wilson’s disease, autoimmune liver disease, viral hepatitis, Gilbers condition, sickle cell anemia, and G6PD deficiency were found to be negative. The TATA-BOX sequence in the UDT1A1 gene was also studied. The patient was managed on a daily dose of phenobarbitone (30 mg/day). Albumin and serum bilirubin levels were monitored weekly for the first month and checked once per month thereafter with a timely assessment of liver enzymes based on the patient’s history of hyperbilirubinemia dating back to childhood and reaction to phenobarbitone, the patient's CNS type II was concluded. Patients’ bilirubin levels were targeted between 7 and 12 mg/dL throughout her pregnancy (Figure 3).

She delivered a healthy baby boy (weight: 2118 g and APGAR score: 8 and 9 at 5 and 10 minutes, respectively) at 37 weeks six days. Bilirubin levels were the same in the mother's blood and cord blood. Post delivery total bilirubin levels raised to 9.6 mg/dL, indirect bilirubin to 8.7 mg/dL, and albumin to 3.2 mg/dL. Infants bilirubin was 3189 mg/dL and albumin was 3500 mg/dL. The cases were managed by a multi-disciplinary team of hematologists, pediatricians, and obstetricians. The infant was subjected to phototherapy (three days), post which his bilirubin decreased to 180 mg/dL and albumin remained steady with no requirement of any further treatment.

## Discussion

**Figure 4:**
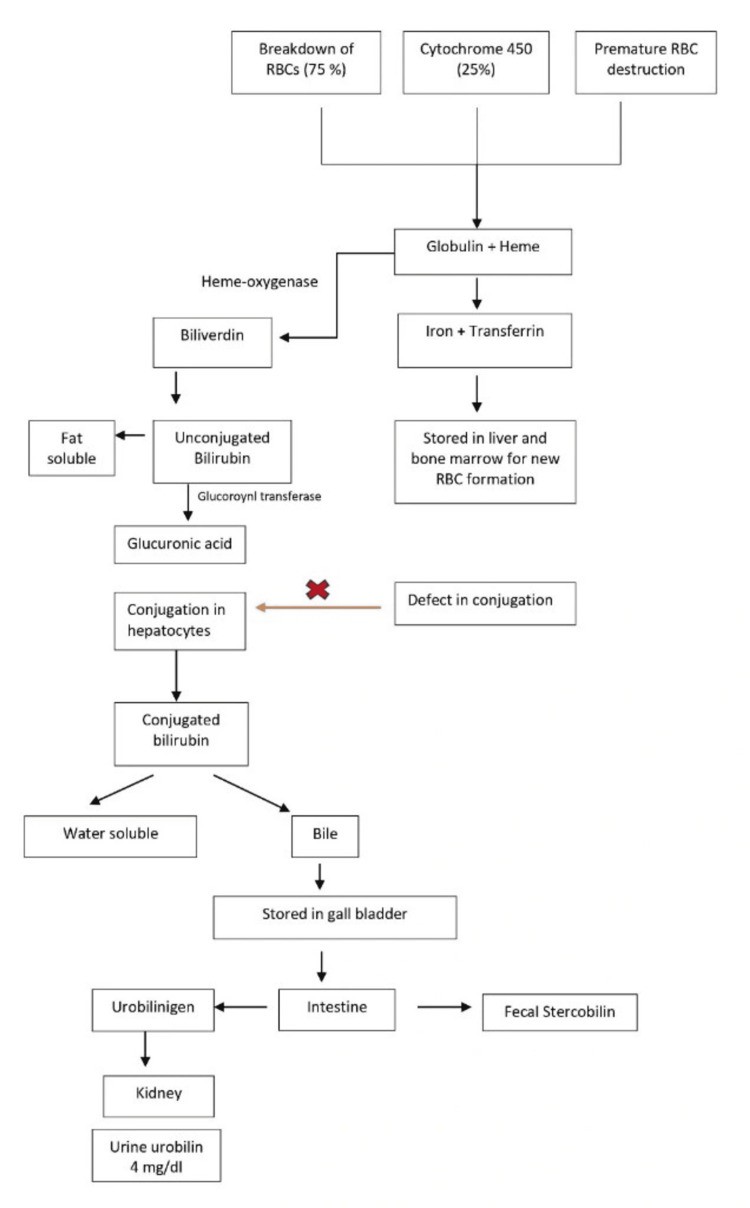
Metabolic pathways of bilirubin metabolism Image credits: Sukanya Singh

CNS is a rare genetic condition with clinical presentation of unconjugated hyperbilirubinemia. It is an autosomal recessive disease, which can be symptomatically observed in neonates but most typically is apparent when individuals with this disease age [2]. Hyperbilirubinemia occurs when bilirubin metabolism is impaired, leading to an increase in both conjugated and un-conjugated bilirubin in the bloodstream (Figure 4).

Types of CNS can also be differentiated into type I and type II for responsiveness to phenobarbital. CNS type II can be differentiated from type I on the basis of reduction of bilirubin levels >25% when managed on phenobarbital therapy. Research has suggested that the gene for enzyme UDPGT, UGT1A1, maps to “2q37,” and mutations linked to both type I and II have been identified, reporting CNS type I as being more severe compared to CNS type II. Pregnant females having CNS can be complicated to manage [1]. This condition affects around 0.6 to one in one million neonates worldwide and is depicted by an accumulation of unconjugated bilirubin in the bloodstream due to impaired conjugation [3]. An increase in bilirubin levels leads to jaundice and can cause a bilirubin build-up in the brain, resulting in kernicterus. CNS type I is critical and presents with severe hyperbilirubinemia in the initial days of life, while type II is less severe and can be treated effectively with phenobarbital [5]. In the absence of conjugation and elimination, unconjugated bilirubin is bound to albumin and exceeds the body’s supply of albumin, raising blood bilirubin levels and can result in its deposit in tissues such as skin, sclera, and brain. This unconjugated bilirubin is unbound and can cross the blood-brain barrier and interact with the nervous tissues, which might result in lasting neurological difficulties in the infant, such as hearing loss, mental retardation, and choreoathetosis.

Hepatic parenchymal damage has also been linked to prolonged exposure to elevated levels of unconjugated bilirubin. Liver cirrhosis is often caused by persistent damage to the liver [6]. Available research studies suggest that CNS type II is not a contraindication for pregnancy, but a swift and coordinated approach from the interdisciplinary connection of gynecologists, pediatricians, hepatologists, hematologists, and genetic experts in these "high-risk pregnancies" can be managed and optimal fetal and maternal outcome be achieved [7]. Diagnosis of intra-hepatic cholestasis of pregnancy must be taken into consideration in conjunction with abnormal liver function tests and linked to a higher incidence of intrauterine mortality, fetal hypoxia, and premature labor. 

Increased bile acid levels, but not alanine transaminase, are indicative of a negative pregnancy outcome. If intra-hepatic cholestasis of pregnancy is detected, hospitalization is required for further evaluation [8]. In the latter trimesters, epigastric discomfort, nausea, vomiting, headache, and malaise are observed. A female with some or all of these symptoms, if not all, pre-eclampsia spectrum of disorders has to be ruled out. Pre-eclampsia during pregnancy may be worsened by elevated transaminase levels. However, these liver abnormalities are seldom presenting symptoms and are not substantially predictive of maternal or fetal outcomes. A screening and preventive strategy can be by regular monitoring of blood pressure (>140/90) and dipstick tests for proteinuria during routine ANC visits with a primary objective to reduce unconjugated bilirubin levels via plasmapheresis and phototherapy.

Phenobarbitol is considered a safe drug in pregnancy and can be used in these cases. Patients whose jaundice negatively impacts their quality of life are treated with phenobarbital, which has the ability to stimulate residual UGT activity. It decreases the serum bilirubin concentration by 25% in CNS type II patients. Plasmapheresis is considered the most efficient method in hyperbilirubinemia crisis, attributed to its strong linkage to albumin, which can directly contribute toward lowering bilirubin levels. Lipid-lowering agents such as clofibrate have also been used for the management of CNS patients, but it is contraindicated in pregnancy. Intense phototherapy is a common treatment modality most effectively used in CNS type I disorder as it cuts down on treatment time, though it has been found less efficient in older children and adults due to the presence of thick skin, higher pigmentation, and decreased body surface area/mass ratio [7]. Orlistat, a lipase inhibitor, when used with calcium phosphate has been found more effective, which is suggestive of absorption of bilirubin photoproducts and its release in bile by tracing the amount of fat excreted in feces, to measure eliminated unconjugated intestinal bilirubin as well. Liver transplantation is the only effective and proven therapy option for CNS type I cases. Serum bilirubin levels decrease rapidly by the transplanted liver. As kernicterus might not always be reversible, prophylactic liver transplantation is advised. Hepatocyte transplantation is an advantageous substitute for liver transplantation, which is done by infusion of normal hepatocytes into the peritoneal cavity or portal vein, claiming a 50% reduction in serum bilirubin levels [9]. However, there are no reports of its use in pregnant patients. An ultrasound-guided IUCT has been conducted in rat fetuses on embryonic day 16, showing the presence of anti-human mitochondria-positive cells being detected in the liver of recipient rats on postnatal day 21 [10]. Gene therapy: A normal UGT1A1 gene might be introduced to potentially treat the genetic abnormality. Also, there are some positive research evidences of mRNA-mediated therapies in animal models paving the way for in-human trials [10,11].

## Conclusions

There are very few case reports on CNS in pregnant females, its diagnosis, therapeutic management, and fetal outcomes. Based on the review of available global literature, we may deduce that pregnancy in CNS patients is still uncommon. Hence, we report this case, its management, and fetal outcomes aiming to contribute to the variability of the clinical research database. We suggest serum bilirubin levels be maintained below 8-10 mg/dL in CNS type II pregnant patients with phenobarbital therapies for safe confinement, along with neurologic follow-up of the children born to these patients. The inclusion of sensitive hearing tests in these children is also strongly recommended, as hearing disorders are reported as one of the most common sequelae of kernicterus; although they can be effectively treated, they are commonly misdiagnosed due to the availability of limited research data. With a prime focus on the safety of the pregnant patient with CNS type II, a positive outcome as a healthy infant serves as a hope for such rare cases impacted by this rare condition association, based on multi-disciplinary clinical management and close monitoring of maternal serum bilirubin.
